# Assessment of Bone Quality using Finite Element Analysis Based upon Micro-CT Images

**DOI:** 10.4055/cios.2009.1.1.40

**Published:** 2009-02-06

**Authors:** Yumie Rhee, June-Huyck Hur, Ye-Yeon Won, Sung-Kil Lim, Myong-Hyun Beak, Wen-Quan Cui, Kwang-Gyoun Kim, Young Eun Kim

**Affiliations:** Department of Internal Medicine, Endocrinology, Yonsei University, Seoul, Korea.; * LCT Orthropaedic Speciality Hospital, Suwon, Korea.; † Department of Orthopedic Surgery, Ajou University School of Medicine, Suwon, Korea.; ‡ Department of Mechanical Engineering, Dankook University, Yongin, Korea.

**Keywords:** Osteoporosis, Vertebral body, Microstructure, Micro-CT, Finite element analysis, Rat

## Abstract

**Background:**

To evaluate the feasibility of a micro-image based finite element model to determine the efficacy of sequential treatments on the bone quality in a rat osteoporosis model.

**Methods:**

Rat osteoporosis and treated osteoporosis models were established with the bone loss, restore and maintain concept. Thirty Sprague-Dawley rats were used in this study. A sham operation or ovariectomy was performed at 20 weeks after birth, which was followed by the respective sequential trials as follows: (1) sham-operation only, (2) ovariectomy only, (3) ovariectomized rats with parathyroid hormone maintenance, (4) ovariectomized rats treated with PTH for 5 weeks and then withdrawal, (5) ovariectomized rats treated with PTH for 5 weeks and then with 17 beta-estradiol, and (6) ovariectomized rats treated with parathyroid hormone for 5 weeks and then treated with zoledronate. The histomorphometry indices were determined using the micro-images from a micro-computed tomogram. Finite element analysis was carried out to determine the mechanical properties (Stiffness and Young's modulus) of the vertebra bodies. The differences in properties between the groups were compared using ANOVA and a Bonferroni's multiple group comparison procedure.

**Results:**

The histomorphometry and mechanical properties were significantly better in groups (3) and (6) than in the groups (1) and (2) (*p* < 0.05). The stiffness (σ_s_) and Young's modulus (E) was highest in group (3) following by group (6).

**Conclusions:**

Finite element analysis based on micro-images provides a useful tool that reflects the changes in micro-structural and mechanical properties of a rat vertebral body with the bone loss, restore and maintain concept.

Conventional technologies, two-dimensional analysis of the histological sections of bone and mechanical tests are unable to determine the subtle changes in bone quality accurately. First, while measurements of the trabecular number and thickness can be quantified from histological sections, it is not possible to determine the anisotropy of trabecular orientation, plate- or rod-like structures and the connectivity of trabecular bone, which is a three dimensional quantity. These three-dimensional structures are likely to play important roles in determining the bone strength. Second, a direct assessment of the mechanical properties of the bone from experiments has large errors and significant uncertainty,^1^,2) mainly because the mechanical load-frame measurements are quite sensitive to friction between the sample and load patterns, and because they depend significantly on the size and shape of the samples.3) Therefore, mechanical testing may not detect small or even large changes in mechanical properties of the bone.

Recently, a high-resolution micro computed tomography (µCT) reconstruction of the trabecular bone was introduced. This technique allows an assessment of the bone microarchitecture in three dimensions. Furthermore, micro-images of the bone obtained from µCT can be converted directly into finite element (FE) models to simulate real mechanical tests, eliminate the experimental artifacts and provide an accurate evaluation of the bone specimen mechanical properties.

Osteoporosis is a skeletal disease caused by an imbalance between bone formation and resorption. Accordingly, some anabolic and anti-resorptive agents, such as parathyroid hormone (PTH) and bisphosphonate, are used to treat osteoporosis patients. One of the treatment principles for osteoporosis requires the replacement of an anabolic agent with an anti-resorptive agent because of the complications associated with the long-term use of anabolic agents.^4^,5) The effects of these treatments are not only reflected by the bone quantity,6) but also by the bone quality, particularly the micro-structural and mechanical properties of bone, even at the regional level, i.e., trabecular but not cortical bone. Therefore, accurate evaluations of these changes in bone quality are important.

This study examined changes in the microstructural and mechanical properties of vertebral bodies in a rat model, which was established along with bone loss, restore and maintain concepts using micro-CT and FE-models. In addition, this study examined whether FE models can determine the changes in the microstructural and mechanical properties as a result of primary and sequential alternative treatments to accurately reflect the therapeutic efficacy differences.

## METHODS

### Animals

Thirty, 5-month-old virgin female Sprague-Dawley rats were housed in a laboratory at 22.2℃ under a 12-h light and 12-h dark cycle and maintained on a Purina laboratory rodent chow diet (Hagribrand Purina Korea Co., Kunsan, Korea) supplemented with 1.17% calcium, 0.77% phosphorous, and 2.5 IU vitamin D/G. All animals were treated according to the guidelines and regulations for the use and care of animals at our university. At the age 5 months, the animals were randomized into the following six groups containing 5 rats each (Table 1).

The sham-operated group (SHAM: Group 1) and ovariectomized group (OVX: Group 2) were treated with the vehicle for 10 consecutive weeks beginning 5 weeks after surgery. All the rats in the remaining four groups were ovariectomized when they were 5 months old, left untreated for 5 weeks, and then treated with the recombinant human parathyroid hormone (rhPTH)(1-84) for 5 weeks. The rats were then divided into the different treatment regimen modes. One group was maintained on rhPTH (1-84) for a further 5 weeks (PTH-M: Group 3), another group was given the vehicle for a further 5 weeks to observe the effects of PTH withdrawal (PTH-W: Group 4). The other two groups were administered anti-resorptive agents, either 17β-estadiol (PTH-E: Group 5) or zoledronate (PTH-Z: Group 6). The rhPTH (1-84) groups were treated daily for 5 days/week with intermittent subcutaneous injections of 100 µg/kg/day. The control group was injected subcutaneously with an equivalent volume of 0.9% normal saline at the same frequency. 17β-estradiol (Sigma Aldrich, St Louis, MO, USA), was injected subcutaneously at 10 µg/kg/day for 5 days/week because it is effective in preventing osteopenia in OVX rats.^7^,8) Zoledronate was injected subcutaneously at 12.5 µg/kg, once per week.9) At the end of the 10 week treatment period, the rats were euthanized, and the vertebrae were harvested and stored in a saline-soaked gauze at -20℃ until analysis.

### µCT Scanning

**Fig. 1:**
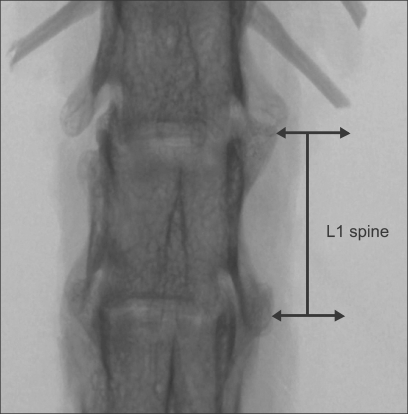
X-ray transmission image.

**Fig. 2:**
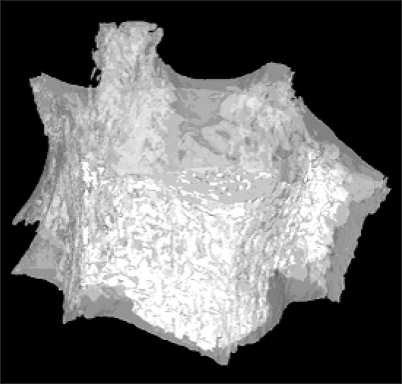
Three dimensional model (L1 Spine).

**Fig. 3:**
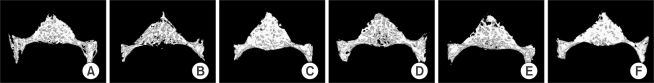
Three dimensional models of the vertebral bodies after treatment. (A) OVX: Ovariectomy group, (B) SHAM: Sham operation group, (C) PTH-M: PTH maintenance group, (D) PTH-W: PTH withdraw group, (E) PTH-E: PTH following estradiol group, (F) PTH-Z: PTH following zoledronate group, PTH: Parathyroid hormone group.

**Fig. 4:**
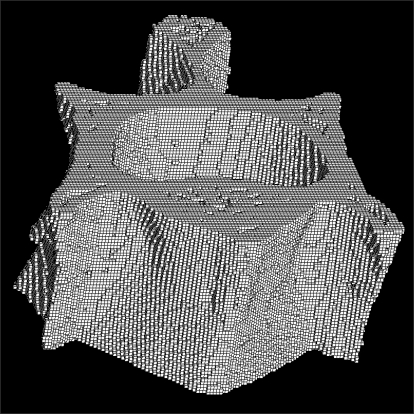
Hexahedral mesh model.

Whole L1 vertebrae were placed in a φ14 mm sample holder in the cranial-caudal direction and scanned using a high-resolution µCT system (Skyscan 1072, SKYSCAN, Kontich, Belgium) at a spatial resolution of 21.31 µm (Voxel dimension) 1,024 × 1,024 pixel matrices (Fig. 1).10) After scanning, 2D the image data was transferred to a workstation, and a 3D reconstruction was performed (Fig. 2).11) The bone tissue was then segmented from the marrow using a global thresholding procedure. From the obtained consecutive micro tomographic slice images, the trabecular bone was extracted from the vertebral body as a volume of interest (VOI) by mapping along with the margin between the cortical shell and trabeculae (Fig. 3) using ANT software (SKYSCAN, Kontich, Belgium). In order to limit the computational requirements for FE analyses, the µCT voxel data was resampled at an isotropic size of 63 µm before converting the three-dimensional bone volume directly into hexahedron-based FE meshes (Fig. 4). Using the built-in software (TomoNT, SKYSCAN, Kontich, Belgium) in the µCT scanner, the following three-dimensional structural parameters were calculated: the bone volume fraction (BV/TV: fraction of trabecular bone per total volume), trabecular number (Tb.N: number of trabeculation per mm), trabecular thickness (Tb.Th: trabecular thickness), trabecular separation (Tb.Sp: distance of each trabeculation), bone surface to volume ratio (S/V: fraction of the total surface area of trabeculation for the total volume), structure model index (SMI: index referring to the plate-like or rod-like structure of trebeculation), and the degree of anisotropy (DOA: index referring to the direction of trabeculation).

### Finite Element Analysis for Mechanical Properties

The reconstruction images of the whole L1 vertebral bodies were converted to micro-finite element (µFE) models by converting the voxels (size 63.8 × 63.8 × 63.8 µm) representing the bone tissue to equally shaped 8-node brick elements using the hexahedron meshing technique.12) A specified threshold level was chosen to ensure the best possible agreement between the BV/TV in the histomorphometry and BV/TV_E_ (element volume fraction) in the FE-model during conversion. For the coarser FE-models, this thresholding procedure caused the loss of the trabecular connection. This resulted in unconnected bone parts that were removed because they did not contribute to the stiffness. The FE-model reconstruction reflects the bone microstructure that modulates the element number to the same BV/TV and BV/ TV_E_.13) The voxel conversion formula was as follows:

BV/TV = BV/TV_E_ = EN/T_N_

(BV/TV: bone volume fraction, BV/TV_E_: element volume fraction, E_N_: element number, T_N_: total number)

**Fig. 5:**
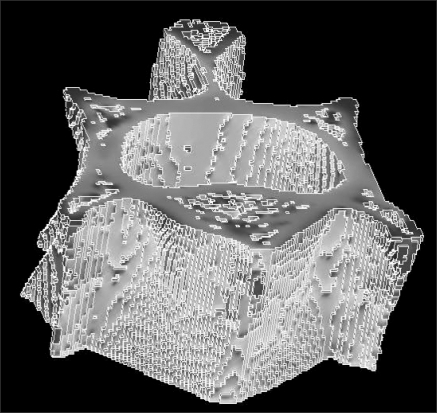
Finite element analysis model.

For all models, the element material properties were assumed to be isotropic, linear elastic, and uniform with a tissue Young's modulus of 1 GPa and a tissue Poisson's ratio of 0.3. The boundary conditions for the FE model were used to represent the situation in a compressive-test setup with a 1%-strain level, where the displacements in the z-direction were unconstrained in the bottom face with all other faces of the cube constrained. The FE-problems for the hexahedron models were solved using ANSYS ver. 9.0 (Ansys Inc, Canonsburg, PA, USA) (Fig. 5). Therefore, the vertebral model is in a state of uniaxial stress at the apparent level. The mechanical parameters calculated were the stiffness (σ_s_) and elastic modulus (E).

### Statistic Analysis

All the data is expressed as the mean ± SD. SPSS ver. 10.0 was used for statistical analysis. The differences in the various histomorphometry indices and mechanical properties between the groups were compared using ANOVA and a Bonferroni's multiple group comparison procedure. *p* < 0.05 were considered significant. On the other hand, linear regression analysis was performed on the groups showing a significant difference in order to determine which histomorphometry index best accounted for the changes in mechanical properties.

## RESULTS

**Fig. 6:**
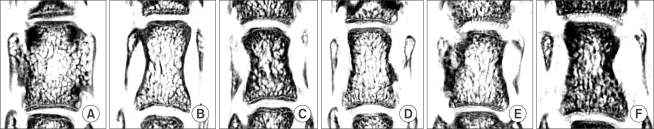
Two dimensional images (sagittal view). (A) OVX: Ovariectomy group, (B) SHAM: Sham operation group, (C) PTH-M: PTH maintenance group, (D) PTH-W: PTH withdraw group, (E) PTH-E: PTH following estradiol group, (F) PTH-Z: PTH following zoledronate group, PTH: Parathyroid hormone group.

Fig. 6 shows typical 2D coronal images of L1 in each group obtained by µCT imaging, which represents the different microstructural patterns of the trabecular bone from bone loss to bone restoration. Tables 2 and 3 show the descriptive statistics of the 2D and 3D structural indices, respectively, as well as the mechanical properties calculated from calculated from finite element analysis (FEA). In the overall view, there were significant differences in both structural and mechanical indices in the PTH-M and PTH-Z groups compared with the SHAM and OVX groups (*p* < 0.05).

### Histomorphology Index

The patterns of trabecular bone loss were observed in the SHAM and OVX groups. Compared with the SHAM group, the OVX group showed decreases in the BV/TV and Tb.N (37.08% and 21.59%, respectively), as well as increases in the Tb.Sp, S/V, and SMI (47.56%, 22.53% and 68.55 %, respectively) (*p* < 0.05) (Table 2, 4). A comparison of the PTH treatment groups with the SHAM group revealed only the PTH-M and PTH-Z to show significant increases in Tb.Th (34.75% and 31.03%). The other PTH-W and PTH-E showed no significant differences (Table 2, 4). However, compared with the OVX group, the PTH-M and PTH-Z showed significant increases in Tb.Th, BV/TV and Tb.N (49.41% and 45.29%, 87.93% and 94.07%, ns and 20.35%, respectively), and decreases in Tb.Sp, S/V and SMI (33.20% and 37.10%, 30.87% and 31.26%, 48.44% and 48.29%, respectively). There were no significant differences in any structural index between the PTH-W and PTH-E groups (Table 2, 4). Compared with the PTH treatment groups, prominent treatment efficacy was observed in the PTH-M and PTH-Z groups, which showed increases in Tb.Th and BV/TV, and decreases in S/V (*p* < 0.05) (Table 2, 4).

### Mechanical Properties

The mechanical properties, σ_s_ and E, were determined from the micro-images using Ansys 7.0 (Ansys Inc., Canonsburg, PA, USA). The mechanical properties of all PTH groups were higher than those of the SHAM and OVX groups. In particular, the mechanical properties of the PTH-M and Z groups were much higher than those of the SHAM and OVX groups. The OVX group showed lower σ_s_ and E values (3.10% and 18.08%) than the SHAM group but without significance (Table 3). A comparison of the SHAM group with each of the PTH treatment groups showed that the PTH-M and PTH-Z groups had significantly higher σ_s_ and E values (SHAM: 31.01% and 63.38%, PTH-M: 29.83% and 70.14%, PTH-Z: 14.29% and 27.07%), but the PTH-W and PTH-E groups showed similar mechanical properties. In addition, the PTH-M and PTH-Z groups had higher elastic moduli than the PTH-E and PTH-W groups (*p* < 0.05).

### Linear Regression Analysis

In order to observe the relationship between the histomorphometry indices and the mechanical properties during the course of bone loss through to the recovery of bone loss, the OVX group was combined with the PTH-M and Z groups and examined by linear regression analysis. Linear regression analysis showed that Tb.Sp, Tb.Th, SMI, S/V, and BV/TV could explain 49.2%, 70.1%, 73.6%, 79.2%, and 80.0% of the change in stiffness in the PTH-M group, respectively, compared with the OVX group. On the other hand, DOA and Tb.N could only account for 0.1% and 28.7% of the changes in σ_s_, which suggests that these relationships were not significant. With the exceptions DOA and Tb.N, the other microstructural indices accounted for the improvements in E (Table 4). A comparison of the PTH-Z group with the OVX group showed that Tb.Sp, SMI, Tb.Th, BV/TV, and S/V could explain 55.2%, 73.8%, 77.8%, 78.1%, and 80.9% of the change in σ_s_, respectively, whereas the relationships between DOA or Tb.N and σ_s_ were not significant. Similarly, with the exception of DOA, the other variables accounted for the improvements in E (*p* < 0.05) (Table 4). 

## DISCUSSION

Given the current concept of the NIH Consensus Development Panel,14) the bone quality contributes to the bone status, and includes the bone mass, bone microarchitecture, bone turnover, bone microdamage, and bone biomechanical properties. This study focused on the bone microarchitecture and biomechanical properties for a therapeutic evaluation because the changes in the bone microarchitecture and bone biomechanical properties reflect the level of bone turnover and bone microdamage.

Recently, high resolution µCT was developed to assess the bone microarchitecture in two and three dimensions. This technique was developed to quantify the 3D structural parameters that define the cancellous bone microarchitecture in animal models and humans. Moreover, based on the µCT micro-images, the micro finite element models were reconstructed to simulate the mechanical tests. Hence, the mechanical properties of the bone were obtained from FEA.

Previous studies reported that the experimental tests used to determine bone biomechanical properties have large errors that are caused by the small sample sizes, shape, geometry, loading conditions, storage, and experimental environment.^15^-18) For example, the Young's modulus and strength of bone will generally increase after the sample is dried but the toughness will decrease.^19^,20) For these reasons, in order to avoid the limitations of experimental testing, this study used the µFE results rather than the experimental test results, because the structural indices investigated do not provide information on the bone tissue parameters or experimental artifacts. By using the µFE results, it was possible to determine the individual contributions made by the structural indices to the bone stiffness without confusion. The FE model can provide insights into the structure-functional relationships of bone.

Overall, a change in trabecular bone loss, restore and maintain can be observed from the micro-CT images. In terms of the pattern of bone loss, the OVX group showed a large decrease in BV/TV and Tb.N, and a significant increase in S/V, SMI, and Tb.Sp, compared with the SHAM group. However, there were no significant differences in Tb.Th or DOA between these two groups. It is possible that unaltered the Tb.Th of the ovariectomized bone tissue is caused by a mechanism in which the bone attempts to compensate for a loss of mass in order to allow a normal mechanical load. In addition, the DOA is an index that reflects the degree of anisotropy in 3D for the trabecular bone. In humans, changes in the 3D structure of the trabecular bone show a tendency toward anisotropy during aging and the progression of osteoporosis. However, the unchanged DOA in the vertebra of ovariectomized rats was attributed to different loading conditions in humans and quadrupeds.

A comparison of the SHAM group with the other treatment groups showed a residual pattern of the microstructural properties. With the exception of the significant increase in Tb.Th in the PTH-M and Z groups, the residual indices in all of treatment group were similar. However, as reflected by the mechanical properties calculated from FEA and σ_s_, E was significantly higher in the PTH-M and Z groups. This demonstrates that these FEA indices are sensitive enough to reflect the occurrence of subtle changes in the microstructure properties, which is probably an advantage of FEA.

Bone loss restoration caused increases in Tb.Th, BV/ TV, and Tb.N, but decreases in Tb.Sp, S/V and SMI in the PTH-M and Z groups. However, all the mechanical indices increased.

Bone strength is often used as a central predictor of the fracture risk. BV/TV, as an index that reflects the amount of bone mass, can best explain the 80% change in stiffness and 60% change in elastic modulus during the course of bone loss through to restoration. Moreover, Tb.Sp, Tb.Th, SMI and S/V could better explain the changes in the mechanical properties, reaching a range of 50% and 80%, as shown in Table 3.

The use of FEA based on the micro-CT images and microstructural indices allows the changes in the microstructural and mechanical properties of a rat vertebral body as well as the amount of bone loss, bone remaining and bone restored to be differentiated. This method can be used to accurately reflect the differences between the therapeutic efficacies of different treatments in small animal models.

## Figures and Tables

**Table 1:**
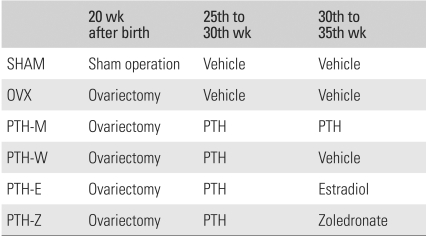
Treatment Methods for Each Group

SHAM: Sham operation group, OVX: Ovariectomy group, PTH-M: PTH maintenance group, PTH-W: PTH withdraw group, PTH-E: PTH following estradiol group, PTH-Z: PTH following zoledronate group, PTH: Parathyroid hormone group

**Table 2:**
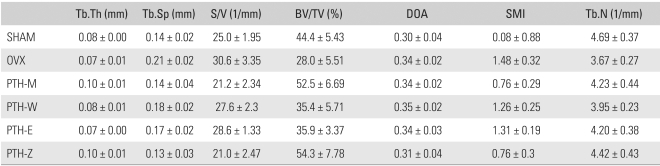
Histomorphometry Indices of the L1 Vertebral Body Trabecular Bones

Tb.Th: Trabecular thickness, Tb.Sp: Trabecular separation, S/V: Surface to volume ratio, BV/TV: Bone volume fraction, DOA: Degree of anisotropy, SMI: Structure model index, Tb.N: Trabecular number, SHAM: Sham operation group, OVX: Ovariectomy group, PTH-M: PTH maintenance group, PTH-W: PTH withdraw group, PTH-E: PTH following estradiol group, PTH-Z: PTH following zoledronate group, PTH: Parathyroid hormone group

**Table 3:**
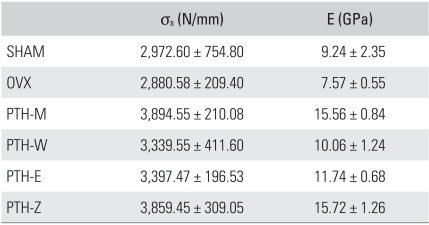
Mechanical Properties of the L1 Vertebral Bones

SHAM: Sham operation group, OVX: Ovariectomy group, PTH-M: PTH maintenance group, PTH-W: PTH withdraw group, PTH-E: PTH following estradiol group, PTH-Z: PTH following zoledronate group, PTH: Parathyroid hormone group

**Table 4:**
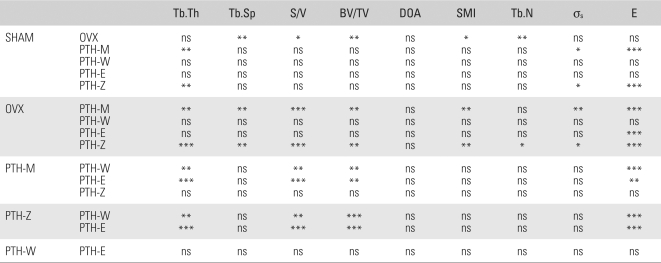
Statistics of All Groups

Tb.Th: Trabecular thickness, Tb.Sp: Trabecular separation, S/V: Surface to volume ratio, BV/TV: Bone volume fraction, DOA: Degree of anisotropy, SMI: Structure model index, Tb.N: Trabecular number, SHAM: Sham operation group, OVX: Ovariectomy group, PTH-M: PTH maintenance group, PTH-W: PTH withdraw group, PTH-E: PTH following estradiol group, PTH-Z: PTH following zoledronate group, PTH: Parathyroid hormone group, ns: *p* > 0.05, ^*^: *p* < 0.05, ^**^: *p* < 0.01, ^***^: *p* < 0.001
